# A SERUM PROTEIN SIGNATURE OF APOE GENOTYPES IN CENTENARIANS

**DOI:** 10.1093/geroni/igz038.2316

**Published:** 2019-11-08

**Authors:** Stefano Monti, Stefano Monti, Paola Sebastiani, Anastasia Gurinovich, Toshiko Tanaka, Lori L Jennings, David J Glass, Thomas T Perls

**Affiliations:** 1 Boston University, Boston, Massachusetts, United States; 2 Division Of Computational Biomedicine, Boston University School of Medicine, Boston, Massachusetts, United States; 3 Department of Biostatistics, Boston University School of Public Health, Boston, Massachusetts, United States; 4 Bioinformatics Program, Boston University, Boston, Massachusetts, United States; 5 Translational Gerontology Branch, National Institute on Aging, Baltimore, Maryland, United States; 6 Novartis Institutes for Biomedical Research, Cambridge, Massachusetts, United States; 7 Geriatrics Section, Department of Medicine, Boston University School of Medicine and Boston Medical Center, Boston, Massachusetts, United States

## Abstract

The discovery of treatments to prevent or delay Alzheimer’s disease is a priority. The gene APOE is associated with cognitive change and late onset Alzheimer’s disease, and epidemiological studies have shown that the e_2 allele of APOE has a neuroprotective effect, and it is associated with increased longevity. We correlated APOE genotype data of 222 New England Centenarian Study participants, including 79 centenarians, 84 centenarian offspring and 55 carriers of APOE e_2, with aptamer-based serum proteomics (SomaLogic technology) of 4783 human proteins corresponding to 4137 genes. We discovered a signature of 16 proteins that associated with different APOE genotypes, and replicated the signature in 3 independent studies. We show that the protein signature tracks with gene expression profiles in brains of late onset Alzheimer’s disease vs. healthy controls. Finally, we show that seven of these proteins correlate with cognitive function changes. Therefore, targeting APOE e_2 molecularly may preserve cognitive function.

